# Supplementary material for: Ventilator-associated pneumonia among ICU patients in WHO Southeast Asian region: A systematic review
Source: PLoS One. 2021 Mar 9;16(3):e0247832. doi: 10.1371/journal.pone.0247832 (PMC7942996; doi:10.1371/journal.pone.0247832)
Supplement: S1 File — (PDF) [file pone.0247832.s002.pdf]

Supplementary Content

Search strategy

Date of search= 22nd September 2020

For PubMed:

((((Incidence OR Prevalence OR VAP rate OR Epidemiology OR Frequency OR "Microbiology"[Mesh])) AND ((((((("Respiration, Artificial"[Mesh]) OR (ventilator acquired)) OR (Ventilator-associated)) OR (ventilator associated)) AND ("Pneumonia, Bacterial"[Mesh])) OR (((VAP) OR (Early VAP)) OR (Late VAP))) OR ("Pneumonia, Ventilator-Associated"[Mesh]))) AND (((((((((((Bangladesh) OR (Bhutan)) OR (India)) OR (Maldives)) OR (Nepal)) OR (Indonesia)) OR (Thailand)) OR (Srilanka)) OR (Myanmar)) OR (Timor-Leste)) OR (South Korea))) AND ((((((((((((((((((((((((((Cohort design) OR Cohort stud*) OR Cohort studies) OR Cross-sectional design) OR Cross-sectional stud*) OR Cross-sectional studies) OR Epidemiologic stud*) OR Epidemiologic studies) OR Longitudinal design) OR Longitudinal stud*) OR Observational stud*) OR Population stud*) OR Prospective design) OR Prospective stud*) OR Prospective studies) OR Prospective design) OR Prospective stud*) OR Prospective studies) OR Retrospective design) OR Retrospective stud*) OR Retrospective studies)) AND ((humans[Filter]) AND (english[Filter]) AND (2000:2020[pdat]))

For Embase:

#21. #9 AND #17 AND #18 AND #19 AND [english]/lim AND [humans]/lim AND [1-1-2000]/sd NOT [23-8-2020]/sd

#20. #9 AND #17 AND #18 AND #19

#19. #10 OR #11 OR #12 OR #13 OR #14 OR #15

#18. #4 OR #5 OR #6 OR #7

#17. #1 OR #8 OR #16

#16. #2 AND #3

#15. 'longitudinal study'/exp

#14. 'prospective study'/exp

#13. 'retrospective study'/exp

#12. 'case control study'/exp

#11. 'cross-sectional study'/exp

#10. 'cohort analysis'/exp

#9. 'bhutan' OR 'bangladesh' OR 'nepal' OR 'india' OR 'maldives' OR 'myanmar' OR 'thailand' OR 'sri lanka' OR 'timor-leste' OR 'indonesia' OR 'south korea'

#8. ventilator AND associated AND pneumonia

#7. epidemiology

#6. 'infection rate'/exp

#5. 'incidence'/exp OR incidence

#4. 'prevalence'/exp OR prevalence

#3. 'bacterial pneumonia'/exp

#2. 'artificial ventilation'/exp

#1. 'ventilator associated pneumonia'/exp
