# Supplementary material for: Ventilator-associated pneumonia among ICU patients in WHO Southeast Asian region: A systematic review
Source: PLoS One. 2021 Mar 9;16(3):e0247832. doi: 10.1371/journal.pone.0247832 (PMC7942996; doi:10.1371/journal.pone.0247832)
Supplement: Attachment — Submitted filename: Response to reviewers.docx [file pone.0247832.s003.docx]

February 11, 2021.

Dr. Joerg Heber

Editor-in-Chief

*PLOS ONE journal* (open access)

We thank you for the time, effort and consideration you have put into our manuscript (PONE-D-20-34750) entitled " Ventilator-Associated Pneumonia Among ICU patients in WHO Southeast Asian Region: A systematic review ".

We would also like to express our utmost gratitude to the reviewer for his/her time and their valuable suggestions. Please find our response to the comments below:

**Editors comments:**

We thank the editor for the time and valuable feedback.

1.Comment: Please ensure that your manuscript meets PLOS ONE's style requirements, including those for file naming. The PLOS ONE style templates can be found at.

Response: We have checked our revised manuscript as per PLOS ONE’s style requirements, including those for file naming and edited if needed.

2.Comment: We note that Figure 2 in your submission contains map images which may be copyrighted. All PLOS content is published under the Creative Commons Attribution License (CC BY 4.0), which means that the manuscript, images, and Supporting Information files will be freely available online, and any third party is permitted to access, download, copy, distribute, and use these materials in any way, even commercially, with proper attribution. For these reasons, we cannot publish previously copyrighted maps or satellite images created using proprietary data, such as Google software (Google Maps, Street View, and Earth). For more information, see our copyright guidelines:

Response: Since, we are unable to obtain permission from the original copyright holder to publish these figures; have decided to remove the figure.

**Reviewer**

We thank the reviewer for the time and for the valuable feedback.

Reviewer response to answers:

1. Is the manuscript technically sound, and do the data support the conclusions?

The manuscript must describe a technically sound piece of scientific research with data that supports the conclusions. Experiments must have been conducted rigorously, with appropriate controls, replication, and sample sizes. The conclusions must be drawn appropriately based on the data presented.

Reviewer #1: Partly

Reviewer #2: Partly

Response: We have tried to address this comment by adding points in our revised manuscript in the conclusion section in Page no 33.

2.Has the statistical analysis been performed appropriately and rigorously?

Reviewer #1: N/A

Reviewer #2: No

Response: Our review article only qualitatively assessed the incidence, mortality and causative organisms of Ventilator Associated Pneumonia with no need of any statistical analysis.

3.Have the authors made all data underlying the findings in their manuscript fully available?

Reviewer #1: Yes

Reviewer #2: Yes

Response: Thank you for your positive response.

4. Is the manuscript presented in an intelligible fashion and written in standard English?

PLOS ONE does not copy edit accepted manuscripts, so the language in submitted articles must be clear, correct, and unambiguous. Any typographical or grammatical errors should be corrected at revision, so please note any specific errors here.

Reviewer #1: Yes

Reviewer #2: Yes

Response: Thank you for your positive feedback.

**Reviewer 1 comments:**

1.Comment: In this systematic review of the literature on ventilator associated pneumonia in the South East Asian Region the Authors aimed to describe incidence, mortality and pathogens in order to implement strategies to prevent and control this disease. Twenty-four papers were selected following Prisma recommendations. VAP incidence range between 2.13 and 116 per thousand days. Mortality range from 16.2% and 74.1%. Acinetobacter spp, Pseudomonas aeruginosa, Klebsiella pneumoniae, Staphylococcus aureus and Enterococcus species are frequently involved. The Authors suggest different strategies to prevent VAP. This topic is relevant and interesting especially in this part of the world but several shortcomings need to be addressed:

Response: Thank you for your encouraging comments.

2.Comment: Introduction: - Include a specific reference line 41-42

Response: A specific reference line in 41-42 is added as highlighted in the introduction section Page no 3 of revised manuscript.

3.Comment:Methods:
a. Specify whether a registration has been made to PROSPERO or to another international registry for systematic reviews. Is essential

Response: We have mentioned our registration details under search strategy subsection of materials and methods section as highlighted in page no.6

b. Outcome are very essentials and maybe a proper research and description of risk factors should be included to increase the relevant of the study

Response: Yes indeed, outcomes related to description of risk factor is important but according to our inclusion criteria only studies reporting incidence, mortality and micro-organisms were included (page no. while most of the studies included in our review have no or limited information on risk factors. So, a brief description of risk factors was avoided but common risk factors from different studies conducted in Asian regions were assessed in discussion (Page no.29).

4.Comment: Result:

a. Do not begin the sentence with an Arabic number line 128 and 130

Response: The changes were made and highlighted in page no 8 and 9.

b. Results about possible risk factors included.

Response: Risk factors were not the outcomes chosen as per in our eligibility criteria. But further risk factors were reviewed briefly in discussion on page no.29(added information is highlighted).

5.Comment: Discussion:

a.  The first paragraph of the discussion should sum the main results of the study: incidence, mortality etiological agents

Response: The first paragraph of discussion was changed as highlighted in page no.28

b. Please include something about the methods of the systematic review: for example, why these outcomes were chosen and not others?

Response: Thank you for your comment. We have included information on the methods used in our systematic review highlighted in the last paragraph of the discussion section in page no. 32.

c. Clarify strengths and limitations of this research!

Response: Thank you for your suggestions. The strengths and limitations of this research was addressed under strengths and limitations section above conclusion highlighted in page no. 32-33

d. Interventions to prevent and control VAP are speculative only and not systematically included in the results, however they are stated in the scope.

Response: Indeed, interventions to prevent and control VAP are speculative only but not included in results because of little or no information in our included articles. So, various intervention programs are mentioned as per the previous literature done in similar settings in the discussion section of page no. 31.

We would once again like to thank the reviewers and editors for their generous and insightful comments to improve the paper.

Sincerely,

Mr. Sanjeev Kharel

Corresponding Author.
